# Social encouragement can influence manual preference in 6 month-old-infants

**DOI:** 10.3389/fpsyg.2014.01225

**Published:** 2014-11-04

**Authors:** Françoise Morange-Majoux, Emmanuel Devouche

**Affiliations:** ^1^Laboratoire de Psychopathologie et Processus de Santé, Institut de Psychologie, UniversitéParis Descartes, France; ^2^Unité de Recherche en Psychiatrie et Psychopathologie, Établissement Public de SantéErasme, France

**Keywords:** handedness, prehension, environmental effects, scaffolding

## Abstract

The purpose of this study was to investigate the influence of social encouragement on infants’ hand movements, in particular on manual preference. Thirty-six infants were observed at 5.5 months. In a first step, their spontaneous manual preference was recorded with an object placed at the midline position. The second step consisted in encouraging infants to use their non-preferred hand by putting the object near that hand and congratulating them. The third step was similar to the first one (object placed at the midline position) except that the infant continued to be congratulated when (s)he used the non-preferred hand for reaching the object. Results showed that half of the infants exhibited a spontaneous manual preference and that a majority of these infants could use their non-preferred hand when verbally encouraged. Moreover, infants showing a left hand preference modified their hand-use more easily than infants showing a right hand preference. Although our findings reveal only a temporary and short-term influence of the social context, results are discussed in light of a socio-cognitive perspective whereby social encouragement can model manual preference, in particular its strength and stability.

Highlights

• At 5.5 months, a manual preference was observed in 47.2% of the infants.

• The preference for the left hand was observed in 35.3% of the infants who presented a manual preference.

• Left-handers change more easily their hand-use than right handers.

## INTRODUCTION

Almost 90% of human adults use their right hand in familiar tasks such as writing, drawing, or throwing a ball (Porac and Coren, 1981). This manual preference is quantified in terms of speed and/or performance, and scores of handedness can vary depending on the specificity of the task considered (Fagard and Marks, 2000). For example, symbolic gestures such as signing or pointing – which appear in the second year of life during the period of linguistic explosion – elicit a stronger degree of predominance of right-handedness than non-communicative manual actions in young children (Bates et al., 1986; Vauclair and Imbault, 2009; Cochet and Vauclair, 2010b; Jacquet et al., 2012). Studies on handedness are organized around two axes. The first aims to qualify specific manual movements, i.e., communicative gestures which appear in the second year of life during the period of linguistic explosion. Within this approach, researchers try to illustrate links between development of language and handedness (Cochet and Vauclair, 2010a; Dellatolas et al., 2012). The second seeks to characterize the development of visuomotor coordination, i.e., speed or anticipation.

Surprisingly, whereas early infant reaching and the onset of handedness around 6 months are often considered as first manifestations of visuomotor coordination with the physical world, few studies have focused on the social dimension of these early asymmetric manual movements although they develop in an interactive social context, during scaffolded activities of everyday life. In this perspective, early infant reaching behavior and the onset of handedness around 6 months are not only the results of early intra-visuomotor coordination with the physical world but also the results of interpersonal auditory-motor coordination. And we can hypothesize that manual laterality develops through adjustments not only to the physical world but also to the social and cultural environment.

The aim of the present study is to evaluate the role of social encouragement in modulating/strengthening manual preference at 6 months. Studies have shown that hand-use preferences appears progressively during the first year of life with unimanual grasping and becomes consistent from 6 months (Michel et al., 1986). They also become stable (Michel et al., 2006) with a predominance of right hand-use (McDonnell, 1979; Ramsay, 1980; Michel and Harkins, 1986; Michel et al., 1986) even though all babies display a brief phase of left hand preference between 4 and 6 months (Morange and Bloch, 1996; Morange-Majoux et al., 2000; Rönnqvist and Domellöf, 2006; Morange-Majoux and Dellatolas, 2010; Morange-Majoux, 2011). However, finer motor skills, i.e., manipulations other than reaching or grasping are not consistently lateralized as early as 6 months (Jacquet et al., 2012), indicating that although the direction of hand preference can stabilize from 6 months of age for some activities, the strength of handedness preference may still vary. Additionally, the spatial context of objects influences hand-use until 6/7 months in such a way that objects positioned in the right hemifield lead to more frequent use of the right hand, while objects positioned in the left hemifield lead to more frequent use of the left hand (Provine and Westerman, 1979; Morange and Bloch, 1996; Fagard, 1998; Sacco et al., 2006; Fagard et al., 2009; Souza et al., 2012).

There is a general consensus today that manual laterality originates in a genetic code (Annett, 1978, 1985, 2002). Twin studies have shown that the frequency of left-handedness in families with at least one left-handed parent increases compared with families with right-handed parents, and no such increase is found in the case of adoption (Briggs and Nebes, 1975; Carter-Saltzman, 1980; Provins, 1997). Recently, Hepper (2013) showed that fetuses exhibited motor lateralized behaviors from 10 weeks of gestation, whereas differential expression of genes in the left and right hemispheres have been identified at 12 weeks’ gestation (Sun et al., 2005). These results suggest that the initial developmental emergence of lateralized behavior is probably under genetic control (Collins, 1977). Nevertheless, handedness coding might not be the result of a simple causal relationship between genetic code and manual laterality. Studies on the genetic determinants of handedness (Medland et al., 2009) indicate that genes account for only 25% of the variance. Thus, since 30 years numerous studies have attempted to show the impact of the environment on handedness and have identified biological or/and social factors as well as interactions between genetic and environmental factors that might orient and strengthen handedness (Fagard, 2001). For example, an excess of testosterone production during fetal life appears to favor left-handedness by slowing down the growth of left hemisphere areas and lessening hemispheric asymmetry (Geschwind and Galaburda, 1985). The fetal position *in utero* (the right ear is mostly against the abdominal wall) could lead to asymmetric vestibular stimulation and *in fine* a right hand orientation at birth (Previc, 1991). Denny (2012) has shown that the side on which a baby is held, the side of the vision field stimulated during feeding, or modeling based on parents’ preferential use of one hand, could favor the use of the right hand in infants. Van der Meer and Husby (2006) have shown that newborn infants move their seen hand more than their unseen hand, demonstrating an effect of postural asymmetry. As for social factors, the specificity of the writing in Japan (Annett, 1985) has been identified as discouraging the use of the left hand. In the same perspective, Faurie and Raymond (2005) have shown that in traditional societies, the proportion of right-handers is lower because social pressure is less strong (Marchant et al., 1995).

While many biological, social, and sensory environmental factors have been explored, the question of the impact of the adult’s influence, on the development of infants’ reaching behavior and handedness has begun to be explored over the two last decades only.

In the context of arm movements, van der Meer and van der Weel (2011) showed that newborn infants move their arms closer to their ear in order to hear their mother’s voice through small loudspeakers that are attached to their wrists. Studies using the still-face paradigm show that the parent’s attitude – interactive *vs* non-interactive – influences the motor activity of the infant. For example several investigators have found that infants exhibit active gesturing of the limbs, increased handling of clothes, touching of face, and sucking during the stressful context of the still-face episode compared to normal play episodes (Murray and Trevarthen, 1985; Toda and Fogel, 1993; Moszkowski and Stack, 2007). Other researchers have found that during the period of maternal unavailability, infants’ pointing behaviors increase in frequency (Fogel et al., 1982) as well as balling up the hand into a fist and making distressed facial expressions (Legerstee et al., 1990; Montirossoa et al., 2012).

In the context of reaching, Darcheville et al. (2004) showed that the number of reach responses was higher when the mother’s voice could be heard and the sensor attached to the infant’s hand was within the reaching place (the reaching place was defined as a virtual location above the infant’s right ear). Additionally, Moszkowski and Stack (2007) found that infants tried to touch their mothers more in the normal interactions than during the maternal still-face episodes. However, Dibiasi and Einspieler (2002) failed to find an interaction between acoustic stimulation and spontaneous arm movements in 12-week-old infants. In this particular study, acoustic feedback was not associated with the infants’ arm movements. Probably, the auditory feedback needs to be both concurrent (i.e., simultaneous with the performed action) as well as contingent (i.e., dependent on the action performed) in order to create a link between perception and action. This was confirmed by Lee and Newell (2013) who investigated the influence of contingent auditory feedback on the development of infant reaching and showed that auditory feedback (mother’s voice or musical tones): (i) increased the amplitude of exploratory arm movements before the onset of reaching; and (ii) increased the number of reaches at the beginning of reaching. These results show that auditory feedback, being a major component of social interaction, stimulates infants’ actions and explorations.

In the context of handedness, the most widely researched field of investigation is that of asymetric holding (Nakamichi and Takeda, 1995; Donnot, 2007; Donnot and Vauclair, 2007; Negayama et al., 2010; Scola and Vauclair, 2010). Numerous studies have revealed that a majority of mothers, even left-handed ones, carry their baby on the left side: this preference for the left hemibody in infant holding occurs in 65–85% of cases. In this position, the right hand side of infants’ bodies is more active (McNeilage, 1987). Scola and Vauclair (2010) found that left-side holding at 2 months was significantly associated with infants’ unimanual preferences. However, the adult’s presence and possible intervention still remain poorly explored. A major contribution on this subject is Michel’s (1992) study showing that the infant’s hand-use tends to match the mother’s hand-use during play and that this matching increases between 7 and 11 months of age. Most of the mothers spontaneously placed the toy at the infant’s midline position over 70% of the time. When they placed the object on one side, it was primarily to the infant’s right side and it elicited right hand-use.

However, the impact of social interaction between adults and infants on early reaching behavior – and consequently of hand preference – has been largely ignore. On the contrary, in most studies on the development of prehension, the infant is either alone with an experimenter or sitting on the mother’s lap and does not receive any feedback such as congratulations or assistance. Motor skills are silently observed. Prehension is seldom studied in an ecological context, where the adult, usually the parent, congratulates the infant when he/she catches an object, and does so all the more for the first attempts. We can hypothesize that babies adjust their manual behavior according to this social feedback. Therefore, the social context and training during the early development of prehension should affect the development of handedness (Porac and Coren, 1986). Yet, the importance of scaffolding – more specifically maternal scaffolding – on infant social development is well-known. Before the infant is able to successfully and autonomously grasp an object, (s)he remains largely dependent on the social environment to access and explore the object world (Penman et al., 1981; Pêcheux et al., 1992; Danis et al., 2000; Bigelow et al., 2004). Based on observations of everyday interactions in the home, Pêcheux et al. (1992) showed that maternal scaffolding plays an important role in the development of attentional abilities in 5- and 8-month old infants. In particular, at 5 months, the infant relied heavily on the mother’s interventions: they focused their attention on the object less when they were alone than when playing with their mother. Although socio-cultural theories differ on the role adults play in supporting development, they all concur on its centrality. The well-known “*zone of proximal development*” defined by Vygotsky (1978) refers to what becomes possible for the infant with the adult’s support. For Valsiner (1987), the adult is an instructor, whereas Bruner (1974) emphasizes that the role of the adult consists mainly in stimulating the infant and Fogel (1993) views the adult as a partner in a process of co-construction. This study addresses the following question: What is the influence of positive verbal feedback on the infant’s prehension, and particularly on the use of a non-preferred hand? We chose to study 5.5-month-old infants, because at this age, infants can reach for objects with one hand but their handedness is not yet well established, not yet automated and could therefore still be modified by environmental factors.

## MATERIALS AND METHODS

### PARTICIPANTS

The participants were 36 5.5-month-old infants (18 girls, 18 boys; *M* = 161 days ± 4.1; range 152–168) recruited from birth lists in the 13th and 14th districts of Paris. Four additional infants were tested but were not included in the final sample due to fussiness. All mothers signed an informed consent form, guaranteeing general anonymous treatment of information.

### APPARATUS

The experiment took place in a laboratory room, at a moment of the day chosen by the parents when the infant would be awake and calm. Infants were seated on the mother’s lap facing a table with his/her hands free to move. The stimuli used in this study were three *Playmobil*© figurines (a construction worker, a school teacher, and a gardener). All the objects were randomly presented, placed on the table in front of the baby, at a reachable distance (the distance between the infant and the object was individually adjusted to enable the baby to reach the object by extending the arm). The object was then gently taken away after the infant had reached for it. The object was replaced after each trial. All trials were videotaped with a camera, placed near the experimenter.

### PROCEDURE

At the beginning, the infant and the female experimenter interacted by handing toys back and forth across the test table until the infant seemed comfortable with the experimenter and the room. This “warm-up” period lasted 1–3 min, after which the test began. The mother was asked to remain silent from this moment on. The experiment contained three steps.

The first step aimed to determine the infant’s preference in hand-use. To assess it, each object was placed at a midline position, between the two hands (about 15 cm from each hand) on a table, at a reachable distance (about 15 cm), in front of the baby. Nine trials were performed. Each trial consisted in presenting one of the stimuli to the infant until (s)he reached for it. The experimenter facing the infant then removed the object and presented a second object until the infant reached for it, and so on across the nine trials.

Because our aim was to test the impact of verbal encouragements on hand-use preference, only infants for whom a preferred hand was determined, went on to the second step. The second step consisted in presenting an object, not at the midline position but slightly displaced toward the non-preferred hand (8–10 cm from that hand) and at 5–7 cm from the midline position). If the infant reached for the object with the non-preferred hand, the experimenter congratulated him/her (“yes, well done”). If the infant reached the object with the preferred hand, this trial was not taken into account and (s)he was not encouraged, and a new trial began. The baby performed three trials with the non-preferred hand (with verbal encouragements at each trial) during step 2.

The third step was similar to the first one (object placed in the midline position) except that the experimenter continued to congratulate the infant when (s)he used the non-preferred hand for reaching the object and remained quiet when the infant used their preferred hand. The three steps lasted on average between 5 and 7 min.

For each baby, the experimenter was the same woman, and presented a smiling engaged face when attending to him/her. For each subject the following measures were obtained: (a) the score of hand-use preference during the first step, (b) the hand used during the first trial of the third step, (c) the proportion of reaching gestures performed by the non-preferred hand during the third step. To compute the score of hand-use preference (a), we used a classical hand-use index [HI = (Right Hand – Left Hand)/(Right Hand + Left Hand); Coryell, 1985; Corbetta and Thelen, 1999]. The criteria used for establishing a manual preference was the following: a left hand preference was based on an HI ≤ -0.50 and a right hand preference on an HI ≥ 0.50 (such a criteria corresponded to at least seven reaching gestures out of nine performed with the same hand).

### STATISTICS

The method used to calculate a confidence interval for the difference between two proportions (DP) is the Newcombe-Wilson method without continuity correction (Newcombe, 1998).

## RESULTS

An HI was computed for all infants on the basis of the nine trials of the first step. The HI was ≥ | 0.50| only for 17 infants among the 36 observed, i.e., (47.2%). Among the 17 infants selected, 11 presented a preference for the right hand (right hand group) and six presented a preference for the left hand (left hand group). The preference for the left hand was thus observed in 35.3% of the infants who presented a manual preference. The proportion of reaching gestures performed by the preferred hand was 90.7% in the left hand group (49 gestures out of 54) and 90.9% in the right hand group (90 gestures out of 99), i.e., more than eight reaching movements out of nine. Subsequent analyses were conducted only on these 17 infants.

During step 2, 14 babies performed the three first trials with their non-preferred hand. One or two additional trials were necessary for only three babies to reach step 2 criteria.

Analysis of the reaching movements produced by infants during the third step included 66 movements in the right hand group (11 infants X 6 gestures) and 36 movements in the left hand group (6 infants X 6 gestures). Analyses revealed a significant decrease of the use of the preferred hand in both groups: from 91 to 39% in the left hand group (*p* < 0.0001), and from 91 to 65% in the right hand group (*p* < 0.0001; see **Table 1**). Moreover, results showed that infants in the left hand group performed significantly less reaching movements with their preferred hand (39%) in the third step, than did infants of the right hand group (65%; DP = 26%, *p*= 0.013, see **Table 1**).

**Table 1:** Number of gestures produced by the preferred hand at steps 1 and 3.

		step 1				step 3								Fisher exact
		*n*	(%)			*n*	(%)			DP (%)		95%CI		*p*-value
Right hand group (*n* = 11)		90/99	91			43/66	65			26		(13.1–38.5)		0.0001
Left hand group (*n* = 6)		49/54	91			14/36	39			52		(32.4–66.9)		0.0001
Comparison of the use of the preferred hand in both group at step 3										26		(6.0–43.8)		0.013

A specific analysis of the *first reaching* gesture following the three reinforcement trials showed that 6 of the 11 infants in the right hand group and four of the six infants of the left hand group, i.e., 10 infants among 17 (58.8%) reached for the object with their non-preferred hand although the object was placed in a midline position. However, the sample size did not allow us the test the significance of this result.

Thus, our study clearly shows that infants tended to decrease the use of their preferred hand (from step 1 to 3) when they were verbally encouraged to use the non-preferred hand, regardless of which hand they initially preferred. However, this tendency is more pronounced for the left hand group than the right hand group.

## DISCUSSION

The first finding of this study is that only 47% of infants have a hand-use preference at this age. The proportion of undetermined hand preference (53% of the babies) confirms previous studies showing that infant hand-use preference emerges from 6 months and is not yet consistent before. However, it is important to note, as Michel et al. (2006) pointed out, that assessing handedness requires using very large sample sizes with multiple assessment periods. Thus, in our study, our goal was to determine a hand-use preference at one moment, not handedness *per se* (which implies long lasting effects).

The second finding of this study is that 65% of the babies have a right hand preference when they are lateralized. This right hand bias is in agreement with previous findings (Carlson and Harris, 1985; Michel and Harkins, 1986; Fagard et al., 2009; Ferre et al., 2010) suggesting the onset of right-handedness starts from 6 months.

Third, we hypothesized that environmental factors such as social feedback have an impact on hand-use preference. This study shows that infants can use their non-preferred hand in a simple prehension task if they are congratulated by an adult. In the first trial of step 3, the hand-use preference can’t be attributed to the spatial context of the object. Thus, results can only be explained by the link between the hand-use and the congratulations and provide some evidence of the importance of interactions between social and biology in learning processes, i.e., social encouragement and operant conditioning, whatever genetic factors are involved. These results show that social interaction can modulate manual preference at least during the experiment. They also support the idea that early reaching behaviors in babies must be considered not only as the onset of visuomotor coordination but also as a socio-cognitive coordination. As Darcheville et al. (2004) pointed out recently, reaching develops in a social interactive context, in the scaffolded activities of everyday life. In line with this perspective, early infant reaching behavior is not only the result of an intra-visuomotor coordination but also the result of interpersonal auditory-motor coordination.

Our experiment lasted a few minutes only, and step 2 involved very few trials. Indeed, our experiment was not designed to study a long lasting effect. This is in itself a limitation of our study but results do nonetheless provide evidence that hand preference can momentarily change with simple social feedback such as congratulations by an unfamiliar adult. In everyday life, parents congratulate and encourage the baby daily when (s)he reaches for an object. One can imagine that this social factor could selectively reinforce the use of one or the other hand. As Fagard (2001) pointed out, the environment could have an intensifying effect on what was only a slightly innate tendency. It is unlikely that mothers deliberately train their infants to use a particular hand. However, during play it is conceivable that the mother’s own handedness may bias the infant’s hand-use (e.g., placing objects near the infant’s right hand) and activate the ipsilateral hand as we have seen (Fagard et al., 2009; Suzuki et al., 2009; Jacquet et al., 2012). Additionally, Michel (1992) has shown that infants match maternal hand-use and that this matching increases with age. Although such parental influence might not affect the direction of the offspring’s handedness, it could affect the degree of lateralization of handedness (Michel, 1992).

The fourth finding of this study is that left-handers switch hands more readily than right-handers. This result is confirmed by many studies with adults and infants (Oldfield, 1971; Michel et al., 2006; Gonzalez et al., 2007). These findings should be compared with Michel’s (1992) results: whereas 54% of the infant’s unimanual hand-use actions match the hand that the mother used, right-handed infants matched more of their mother’s hand actions. Hence, right-handedness seems to be more consistent than left-handedness. For Michel et al. (2006), this is a sign that hemispheric lateralization is better established in right-handers than in left-handers.

Handedness is probably the result of multiple interactions between genetic and environmental factors. If babies have a genetic right hand orientation, our experiment shows that this tendency can be momentarily influenced by the social context, and one could surmise that it could be durably reinforced or impeded by repetitive daily parental scaffolding. Bandura’s (1986) social cognitive theory provides an interesting framework for discussing our results. According to Bandura (1986), the subject is at the heart of a triad of interacting factors: social, behavioral and environmental. This means that, although participants are indeed subjected to the principles of operant conditioning, they also exhibit an intentionality and motivation that will influence the way in which they act. They are simultaneously products and producers of their environment. This model emphasizes the essential role of environment for *acquiring* and *maintaining* behaviors and constitutes a deep process of learning in the baby. The organism, task, and environmental constraints all interact to construct the evolving formation of this dynamic landscape (Newell, 1986; Newell et al., 2003).

Little is known about the impact of social interaction on handedness whereas few studies have shown that maternal scaffolding impacts on the “urge” or “motivation” of object prehension and manipulation (Penman et al., 1981; Pêcheux et al., 2000). To go further in the understanding of the influence of social context on handedness, it would be of interest to compare the current situation with one in which a disembodied voice congratulates the infant. Moreover, naturalistic observations with longitudinal design should be conducted in order to determine the stability and the strength of social influence on object manipulation and handedness.

## Conflict of Interest Statement

The authors declare that the research was conducted in the absence of any commercial or financial relationships that could be construed as a potential conflict of interest.
